# Bioelectrical Impedance Vector Analysis and Phase Angle on Different Oral Zinc Supplementation in Eutrophic Children: Randomized Triple-Blind Study

**DOI:** 10.3390/nu11061215

**Published:** 2019-05-28

**Authors:** Karina M. Vermeulen, Márcia Marília G. D. Lopes, Camila X. Alves, Naira J. N. Brito, Maria das Graças Almeida, Lucia Leite-Lais, Sancha Helena L. Vale, José Brandão-Neto

**Affiliations:** 1 Post-graduate Program in Health Sciences, Federal University of Rio Grande do Norte, CEP 59012-570 Natal, RN, Brazil; karinavermeulen@hotmail.com; 2 Department of Nutrition, Federal University of Rio Grande do Norte, CEP 59078-970 Natal, RN, Brazil; mariliagdantas@hotmail.com (M.M.G.D.L.); camila_xavieralves@yahoo.com.br (C.X.A.); ludl10@hotmail.com (L.L.-L.); 3 Department of Pharmacy, University of Cuiabá, CEP 78850-000 Cuiabá, MT, Brazil; nairabrito@yahoo.com.br; 4 Department of Clinical and Toxicological Analysis, Federal University of Rio Grande do Norte, CEP 59012-570 Natal, RN, Brazil; mgalmeida84@gmail.com; 5 Department of Internal Medicine, Federal University of Rio Grande do Norte, CEP 59012-570 Natal, RN, Brazil

**Keywords:** body composition, cell membrane, bioimpedance

## Abstract

The parameters derived from bioelectrical impedance, phase angle (PA) and bioelectrical impedance vector analysis (BIVA) have been associated with cell membrane integrity and body cell mass. Zinc is a micronutrient that exerts important structural functions and acts in maintaining cellular functionality. To evaluate cell integrity and body cell mass, PA and BIVA were evaluated in children orally supplemented with zinc at different concentrations. Anthropometric, bioelectrical (resistance and reactance) and serum zinc variables were collected from two randomized, triple-blind, controlled clinical trials. Sampling was composed of 71 children consisting of three groups: a control group who received a placebo and two experimental groups who received oral supplementation of 5 or 10 mg-Zn/day for three months. The three groups presented increases (*p* < 0.001) in the linear height and weight. In the group supplemented with 10 mg-Zn/day, there was an increase in reactance values (*p* = 0.036) and PA (*p* = 0.002), in addition to vector displacement (*p* < 0.001) in relation to the confidence ellipses. An increase in serum zinc concentration was found (*p* < 0.001) in all three groups. Whit this, the supplementation with 10 mg-Zn/day promotes changes in the integrity of the cell membrane associated with the increase in the cellular mass of healthy children.

## 1. Introduction

Bioelectrical impedance (BIA) has been widely used in clinical practice to evaluate the body composition of adults and children. Compared with other methods for this purpose, the BIA has advantages, including safety, low cost, easy-to-use, portability and practicality [1]. The BIA has based on the principle that body tissues behave as an electric circuit in steady state equilibrium, offering opposition to the electric current when it is applied to the circuit. The impedance (Z), the name assigned to this opposition, presents two vectors: resistance (R) and reactance (Xc). R reflects the quantity of intra- and extracellular fluids, and Xc, the quantity of cell mass, the structure and cell membranes functionality. From these vectors, it is possible to calculate the phase angle (PA) and perform the bioelectrical impedance vector analysis (BIVA) [2,3].

PA is related to cell integrity and functionality, being important both in healthy people assessment and in prognosis of diseases because it reflects different electrical properties of the body tissues. It also indicates nutritional status and hydration. Its evaluation is superior to other nutritional, anthropometric and serum indicators in different populations [4,5,6,7,8].

BIVA is useful for clinical purposes because it detects changes in hydration or body composition, as demonstrated by Carrasco-Marginet et al. [9] and Koury et al. [10]. Using BIVA, it is possible to evaluate the patient by direct vector impedance measurements because this method does not depend on equations or models; it is a graphical method with R and Xc corrected for height, which can generate three analyses: individual, follow-up, and groups [3]. 

The plasma membrane has three main functions: coating, protection and selective permeability [11]. Zinc is involved in the integrity, stabilization of structural membranes, protection and cellular functionality, and exerting structural, catalytic and regulatory functions. Thus, zinc acts as a cofactor of several metabolic pathways [12,13]. Studies show that zinc deficiency increases erythrocyte membrane fragility, and compromises platelet aggregation and osmotic protection [11,14,15]. 

The participation of zinc in membrane stability is described in the literature through three mechanisms. First, zinc promotes the association between membrane proteins and cytoskeleton proteins. Second, zinc stabilizes the reduced form of sulfhydryl groups, contributing to the antioxidant protection against the effects of membrane rupture caused by lipid and protein oxidation. Third, zinc preserves the integrity of ion channels, thus acting as an antagonist to the adverse effect of free Ca^+2^ [10,16]. However, to the best of our knowledge, no studies evaluate the influence of different zinc concentrations on cell integrity and functionality using PA and BIVA as the evaluation method. This study aimed to evaluate changes in PA and BIVA in healthy children orally supplemented with zinc at different concentrations.

## 2. Materials and Methods

### 2.1. Study Design and Population

A database search of two clinical trials, randomized, controlled, triple-blind and non-probabilistic was conducted, had as main objective the supplementation of zinc in apparently healthy children. The partial results of these studies were previously published [17,18,19]. These studies were approved by the Ethics Committee of the University Hospital Onofre Lopes (HUOL) by the Federal University of Rio Grande do Norte (UFRN) (protocol numbers 323/09 and 542/11). 

The tests were carried out with different oral zinc supplementations in apparently healthy children (stage of sexual maturation suitable for the age, without acute, chronic, infectious or inflammatory diseases), aged between six and nine years. The children were recruited at four municipal schools located in the east and west of the city of Natal, Rio Grande do Norte, Brazil.

Exclusion criteria were children in pubarche, thelarche or menarche, who had undergone surgery of any kind, or who used vitamin or mineral supplements. An endocrinologist performed these clinical evaluations. 

**Figure 1:**
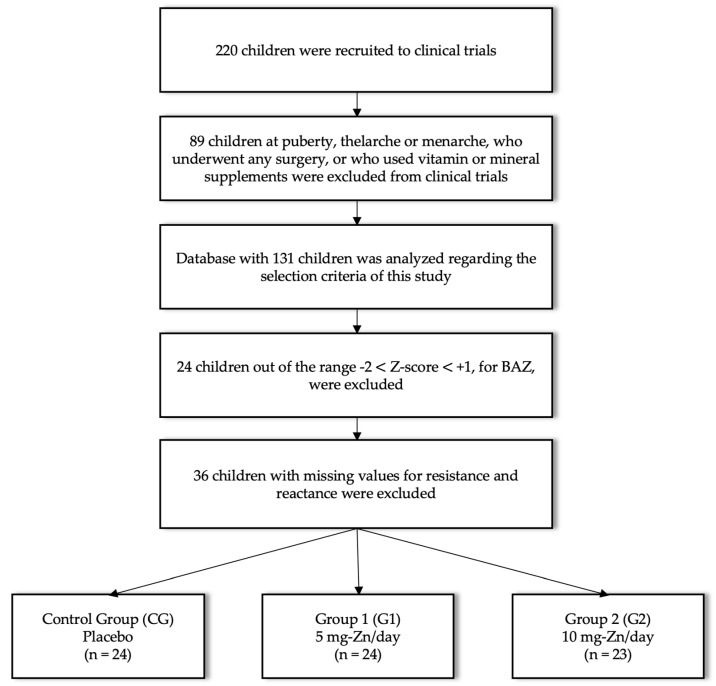
Recruitment flowchart with inclusion and exclusion procedures.

Among the children who comprised this study’s sample, only those eutrophic were included, considering body mass index for age Z-score (BAZ) (Z-scores between −2 and +1) [20]. In addition, children with incomplete information for BIVA implementation were excluded. The values of age (years), weight (kg), height (cm), resistance (Ω), reactance (Ω) and serum zinc (μg/mL) were measured immediately before the beginning of zinc supplementation (T0) and immediately after three months of supplementation (T1). Recruitment, inclusion, and exclusion procedures are described in Figure 1.

### 2.2. Standardization of Clinical Trials

Both clinical trials were performed by the same research group and had the same methodology for data collection and analysis with standardized protocols.

#### 2.2.1. Oral Zinc Supplementation

Stratification of the sample according to the oral zinc supplementation resulted in the formation of three groups: the control group (CG) received a placebo (10% sorbitol, the same vehicle used in the zinc solution); Group 1 (G1) received 5 mg-Zn/day; and Group 2 (G2) received 10 mg-Zn/day. The supplementation period was three months. Zinc was supplied in the form of zinc sulfate heptahydrate (ZnSO_4_·7H_2_O; Merck, Darmstadt, Germany). Each drop of the supplement contained 1 mg of the zinc element. Zinc sulfate heptahydrate acquisition and the oral zinc solution preparation were performed as described by Brito et al. [19]. Those responsible for the children were instructed to add the supplement to water, milk or juice at breakfast.

#### 2.2.2. Assessment of Serum Zinc

Serum zinc was determined by atomic absorption spectrophotometry (SpectrAA-200, Varian, Victoria, Australia). Four milliliters of blood were collected for serum zinc analyses. All processes related to the collection, separation, dilution, and storage of blood for the serum zinc dosage, as well as the calibration of the apparatus, standard serum control, and zinc measurements, were performed as described by Brito et al. [19]. Zinc sensitivity was 0.01 µg/mL, intra-assay coefficient was 2.09% and reference values was 0.7–1.2 µg/mL, according our laboratory evaluation. The clinical examination found no signs or symptoms of zinc deficiency. 

#### 2.2.3. Anthropometric Assessment

The weight and height of the children participating in the trials were measured using an electronic scale (Balmak, BK50F, São Paulo, Brazil) and stadiometer (Sanny, São Paulo, Brazil), respectively. BMI (kg/m^2^) was calculated as the ratio between body weight and height squared. The weight-for-age (WAZ), height-for-age (HAZ) Z-scores and BAZ were calculated using AnthroPlus software v1.0.4 (available at www.who.int/growthref/en/) and ranked according to the growth curves of the World Health Organization for healthy children aged 5–19 years [20]. Trained nutritionists performed anthropometric assessments.

#### 2.2.4. Bioelectric Impedance

The bioelectrical impedance parameters, R (Ω) and Xc (Ω), were obtained using the Quantum II^®^ body composition analyzer (RJL Systems, Clinton Township, MI, USA) with a single, safe and painless electrical frequency (50 kHz). This tetrapolar method was applied with the subject lying supinated. Four self-adhesive spot electrodes were placed: two on the dorsal surface of the right hand and two on the dorsal surface of the right foot, as described by Lukaski et al. [21]. With the parameters obtained by the bioelectric impedance, the percentage of fat-free mass (%FFM) was calculated using the equation proposed by Houtkooper [22], then the PA and BIVA were performed.

### 2.3. Bioelectrical Impedance Vector Analysis

R and Xc data were subsequently used to determine PA and BIVA. The PA was calculated with the formula PA = arc tang (Xc/R) × 180/π [23]. The BIVA results were based on the analysis of normalized R and Xc values for children’s height measurements (R/H and Xc/H in Ω/m).

BIVA charts directly measure the vectors R and Xc. According to the RXc chart, children’s standardized impedance measurements are represented as bivariate vectors with their confidence and tolerance intervals, which are ellipses in the RXc plane. These vectors do not depend on equations. To investigate the differences between groups, we plotted the 95% confidence intervals for the mean impedance difference of the bivariate vectors measured under two conditions for each group [24].

The position and length of the vector provide information on the hydration status, cell mass, and cell integrity. It is an upward or downward displacement of the main axis associated with more or less soft tissue cell mass, respectively. A significant value for the T2 statistic is evidence that the mean vectors of each group are different [24].

### 2.4. Statistical Analysis

Statistical analysis was performed by observing the data distribution using the Shapiro–Wilk test. All quantitative variables presented normal distribution and were expressed as mean (standard deviation), except the age that presented non-normal distribution and was expressed as median (Q1; Q3). Intragroup comparisons were performed using Student’s t-test. 

For BIVA, all analyses were performed using the BIVA software [24]. The mean differences between the impedance vectors in the different supplemented groups were determined using the Hoteling T2 test. Statistical tests were considered significant at 5% (*p* ≤ 0.05).

## 3. Results

With the methodology described, data were collected from 71 children aged 6.2–9.9 years. The sex distribution in each group were: 50% both sexes of the CG; and 58% and 39% female in G1 and G2, respectively.

**Figure 2:**
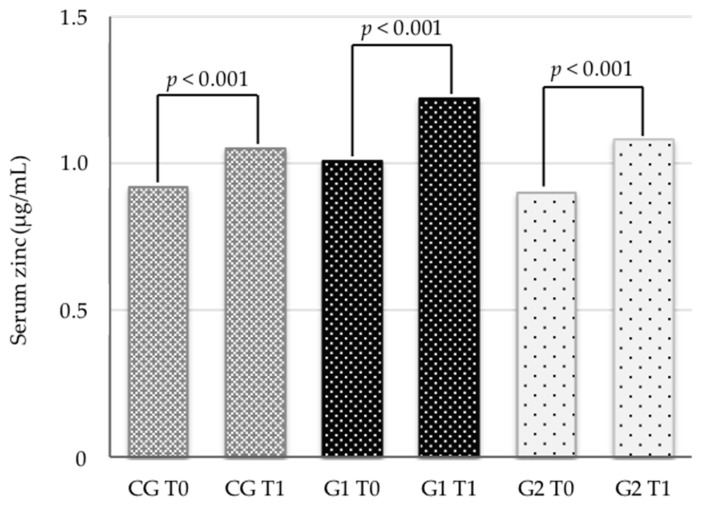
Intragroup comparison of serum zinc before (T0) and after (T1) three months of intervention with different oral supplementation of zinc in eutrophic children.

Concerning serum zinc concentrations, we observed that the children had no apparent zinc deficiency before the intervention (Table 1). There was a significant increase (*p* < 0.001) in serum zinc concentrations in all groups, regardless of supplementation (Figure 2).

Table 2 shows that regardless of the zinc concentration offered, the three groups improved (*p* < 0.001) the linear height and weight, but only the group that received a concentration of 10 mg-Zn/day had an improvement in the values of Xc (*p* = 0.036) and the PA (*p* = 0.002).

**Figure 3:**
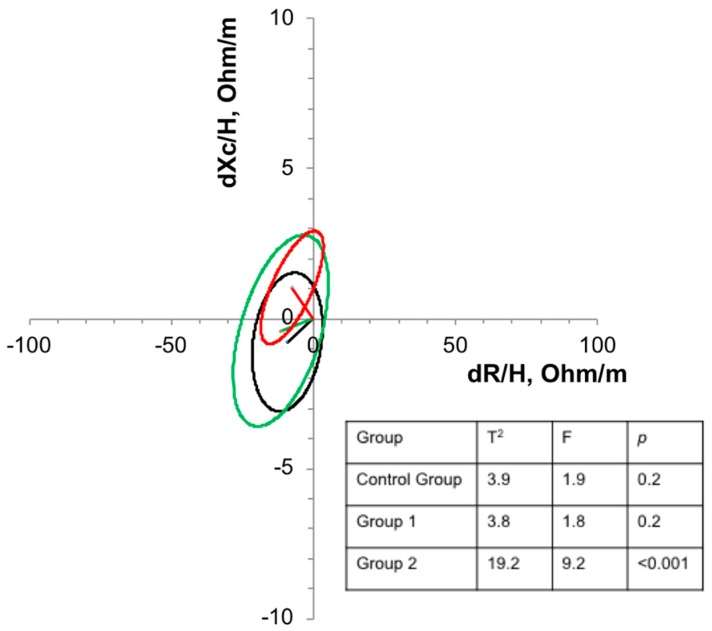
Confidence ellipses of 95% of impedance vectors measured before (T0) and after (T1) three months of intervention with oral supplementation with different concentrations of zinc in eutrophic children. Black ellipse = control group; Green ellipse = Group 1; Red ellipse = Group 2. An upward or downward displacement of the main axis is associated with more or less soft tissue cell mass, respectively.

Regarding the BIVA (Figure 3), only the concentration of 10 mg-Zn/day was enough to promote significant displacement (*p* < 0.001) in relation to the confidence ellipses, indicating a possible increase in the cellular mass in these children.

## 4. Discussion

Our results show that zinc supplementation modified intrinsic factors related to body composition, such as cell integrity and cell mass, even before changes in serum zinc or anthropometric indicators can be detected, as discussed below.

In the population studied, there was an increase in serum zinc over time in all groups, with no differences between them. This study was conducted in a city located on the Atlantic coast, where the supply of food sources rich in zinc is vast. In the study developed by Alves et al. (2016), consumption of energy, protein, fat, carbohydrates, iron and zinc were adequate according to dietary reference intakes by age and sex. Mean zinc intake in these population was 6.00 ± 1.01 mg/ day [25].

Recently, zinc supplementation studies with concentrations ranging from 5 to 50 mg-Zn/day in different infant populations to assess the influence on growth and development have been conducted [26,27,28,29]. In a systematic review, Liu et al. [30] described that zinc supplementation improves growth parameters with potentially stronger effects in children after two years of age. 

The children were in a phase that naturally shows changes in growth. In our study, the anthropometric parameters of WAZ and HAZ did not show differences between the supplemented and control groups. Cho et al. [29], when evaluating children who received 5 mg-Zn/day for six months, also did not observe differences in WAZ and HAZ when compared with the control group. It should be emphasized that the population of our study, although classified as having negative scores at T0, neither demonstrated a deficit in height or weight at the beginning of the study, nor indicated zinc deficiency.

Zinc is essential for the integrity and functionality of cell membranes. Its concentration in the cell membrane can be quite high depending on the cell type and is influenced by the nutritional status of zinc in the organism [31]. In the present study, supplementation with 10 mg-Zn/day significantly increased the values of Xc and PA. Increased PA values are associated with improved cell integrity, lean mass, and the relationship between water distribution in the intracellular and extracellular compartments [32].

Koury et al. [10] found higher PA in adolescents with zinc concentrations in the erythrocyte above the median, concluding that bone age and erythrocyte zinc contribute to PA values in young male soccer players. PA has also been described as a sensitive and useful tool to detect changes in nutritional status in addition to being associated with the clinical prognosis of several diseases [4,5]. Pileggi et al. [8] concluded that PA is a useful tool for detecting nutritional risk in children with osteogenesis imperfecta.

In addition to the increase in Xc and PA in the present study, a significant shift to the upper left quadrant was observed in relation to the confidence ellipses in the group that received 10 mg-Zn/day. 

The use of bioelectric impedance to estimate body composition is a promising methodology [33]. However, one disadvantage is the need to choose equations validated for each specific population [34], since they can be influenced by biological and clinical factors [35,36].

Piccoli et al. [37] proposed that impedance can be plotted in a cartesian plane as a bivariate vector derived from R and Xc, standardized by the height of the individual. Thus, the displacement up or down of the main axis becomes associated with more or less soft tissue cell mass, respectively [24]. 

In their study, Meleleo et al. (2017) concluded that BIVA could provide more reliable details about differences in body composition in competitive and noncompetitive adolescents [38]. This method of evaluation can also be used in other clinical conditions, as described by Juarez et al. (2018), who noted that BIVA might be an option for cachexia in patients with rheumatoid arthritis [39]. Therefore, PA and BIVA can be used together to indicate cellular integrity and hydration without requiring predictive equations.

A strength of this study was the early detection of changes in bioelectrical parameters as a result of zinc supplementation. Based on that, we suggest that PA and BIVA can be used together to indicate cellular integrity and hydration status, respectively, without requiring predictive equations. These findings encourage the replication of this study in other populations. On the other hand, the sample size and the disregard of other micronutrients, such iron, were limitations of this study. The exclusion of 24 participants with missing data and the distribution of the children in three different groups reduced the sample size and may have affected the power of the study. Iron and calcium interact with zinc and may influence bioelectrical parameters. Thus, the nutrition status of other micronutrients must be considered in future studies with a similar aim.

## 5. Conclusions

Oral zinc supplementation with 10 mg/day promoted Xc and PA changes, in addition to a vector shift by BIVA, which was associated with changes in cell membrane integrity and an increase in cell mass in eutrophic children. These original results were observed before changes in serum zinc concentrations or anthropometric indicators.

## Figures and Tables

**Table 1:** Group characterization before oral zinc supplementation with different concentrations (CG = placebo; G1 = 5 mg-Zn/day; G2 = 10 mg-Zn/day).

Group	CG	G1	G2	*p* ^1^
n	24	24	23	--
Age (years)	8.4 (0.5)	8.1 (1.0)	9.1 (0.5)	<0.001
Serum zinc (μg/mL)	0.92 (0.13)	1.01 (0.12)	0.90 (0.11)	0.007
WAZ	−0.15 (0.79)	−0.85 (0.82)	−0.43 (1.05)	0.031
HAZ	0.26 (0.98)	−0.64 (0.82)	−0.39 (1.19)	0.008
BAZ	−0.47 (0.74)	−0.70 (0.68)	−0.27 (0.75)	0.142
Resistance (Ω)	773 (41)	807 (65)	748 (91)	0.017
R/H (Ω/cm)	590.1 (41.7)	654.4 (73.3)	576.0 (87.8)	0.001
Reactance (Ω)	71 (8)	75 (9)	68 (6)	0.008
Xc/H (Ω/cm)	54.3 (7.2)	60.8 (7.9)	52.2 (6.4)	<0.001
Phase angle (°)	5.28 (0.68)	5.34 (0.61)	5.25 (0.64)	0.879
FFM (%)	79.8 (2.8)	78.7 (4.2)	78.8 (3.7)	0.524

WAZ, weight-for-age Z-score; HAZ, height-for-age Z-score; BAZ, Body mass index-for-age Z-score; R/H, Resistance/Height; Xc, Reactance/Height; FFM, fat-free mass. Continuous variables are presented as the means (standard deviations). ^1^ One-way ANOVA.

**Table 2:** Anthropometric characteristics and bioelectric parameters before and after oral supplementation with different concentrations of zinc.

	Control Group			Group 1			Group 2		
Anthropometrics	Before	After	*p* ^1^	Before	After	*p* ^1^	Before	After	*p* ^1^
Weight (Kg)	27.4 (3.3)	27.2 (3.9)	<0.001	22.8 (3.4)	23.4 (3.8)	<0.001	27.1 (4.4)	28.0 (4.5)	<0.001
Height (cm)	131.2 (6.4)	132.6 (6.4)	<0.001	123.8 (6.9)	125.1 (7.0)	<0.001	130.6 (6.8)	132.1 (6.9)	<0.001
BMI (Kg/m²)	15.3 (1.1)	15.4 (1.3)	0.141	14.8 (1.1)	14.9 (1.2)	0.334	15.8 (1.2)	15.9 (1.3)	0.053
WAZ	−0.15 (0.79)	−0.15 (0.89)	0.980	−0.85 (0.82)	−0.85 (0.88)	0.938	−0.47 (1.08)	−0.43 (1.07)	0.152
HAZ	0.26 (0.98)	0.24 (0.99)	0.339	−0.64 (0.82)	−0.65 (0.80)	0.574	−0.39 (1.19)	−0.37 (1.18)	0.316
BAZ	−0.47 (0.74)	−0.46 (0.87)	0.809	−0.70 (0.68)	−0.69 (0.75)	0.970	−0.27 (0.75)	−0.24 (0.75)	0.500
**Bioelectrical**	**Before**	**After**	***p*^1^**	**Before**	**After**	***p*^1^**	**Before**	**After**	***p*^1^**
R (Ω)	772.8 (41.3)	777.7 (50.7)	0.559	806.7 (65.1)	800.0 (71.6)	0.370	748.3 (91.1)	747.2 (101.6)	0.835
R/H (Ω/cm)	590.1 (41.7)	588.3 (56.5)	0.782	654.4 (73.3)	642.9 (83.7)	0.079	576.0 (87.8)	568.6 (93.0)	0.089
Xc (Ω)	71.0 (7.7)	71.9 (9.3)	0.579	75.0 (8.6)	75.0 (6.6)	0.955	67.9 (6.2)	70.0 (7.5)	0.036
Xc/H (Ω/cm)	54.3 (7.2)	54.5 (9.0)	0.863	60.8 (7.9)	60.3 (7.9)	0.737	52.2 (6.4)	53.3 (7.3)	0.163
PA (Ω)	5.28 (0.68)	5.30 (0.58)	0.882	5.34 (0.61)	5.40 (0.49)	0.537	5.25 (0.64)	5.43 (0.68)	0.002
FFM (%)	79.8 (2.8)	79.2 (3.3)	0.065	78.7 (4.2)	78.9 (4.2)	0.478	78.8 (3.7)	78.7 (3.2)	0.558

WAZ, weight-for-age Z-score; HAZ, height-for-age Z-score; BAZ, Body mass index-for-age Z-score; R, resistance; Xc, reactance; H, height; FFM, Fat-free mass. Variables are presented as mean (standard deviation). ¹ Student’s t-test.
